# Supplementary material for: miR-301a-5p Regulates TGFB2 during Chicken Spermatogenesis
Source: Genes (Basel). 2021 Oct 25;12(11):1695. doi: 10.3390/genes12111695 (PMC8621736; doi:10.3390/genes12111695)
Supplement: Supplementary file 1 [file genes-12-01695-s001.zip › genes-1418935-supplementary.pdf]

Supplementary Figures

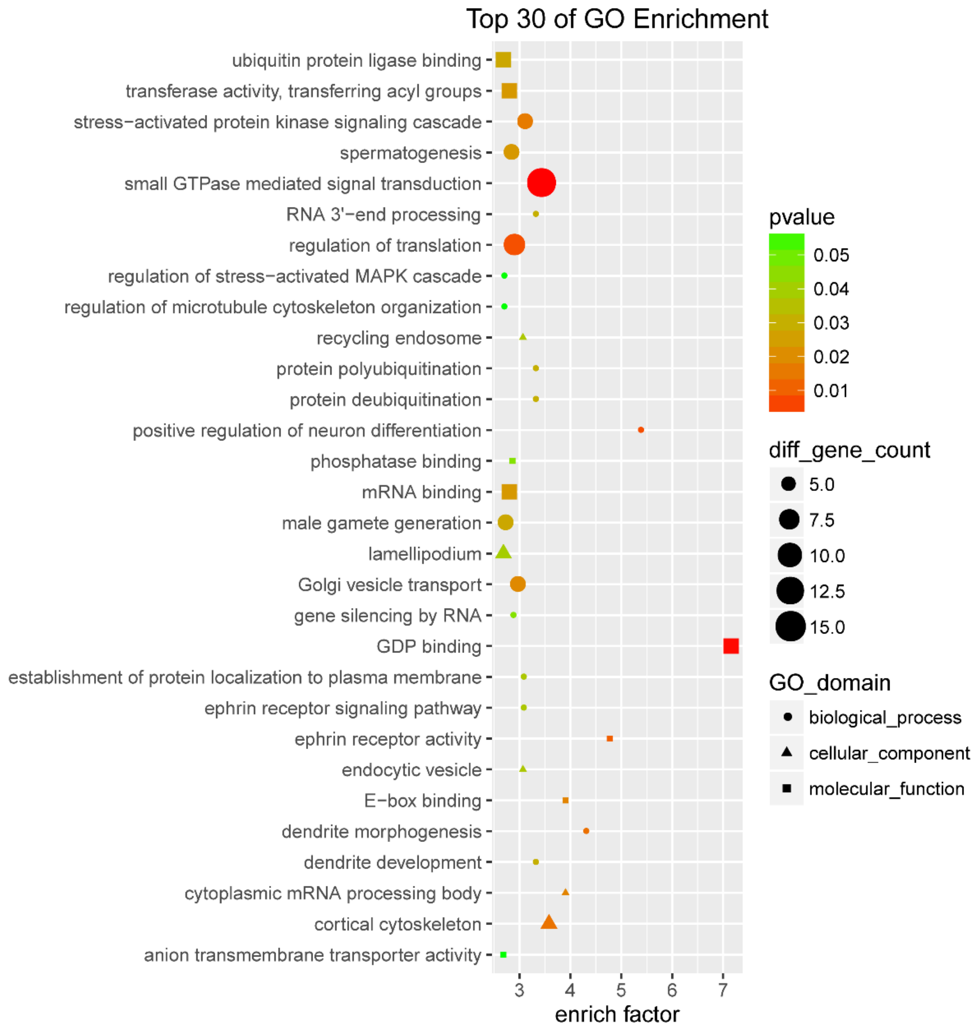

Figure S1. GO terms enrichment analysis of all miRNA target genes.

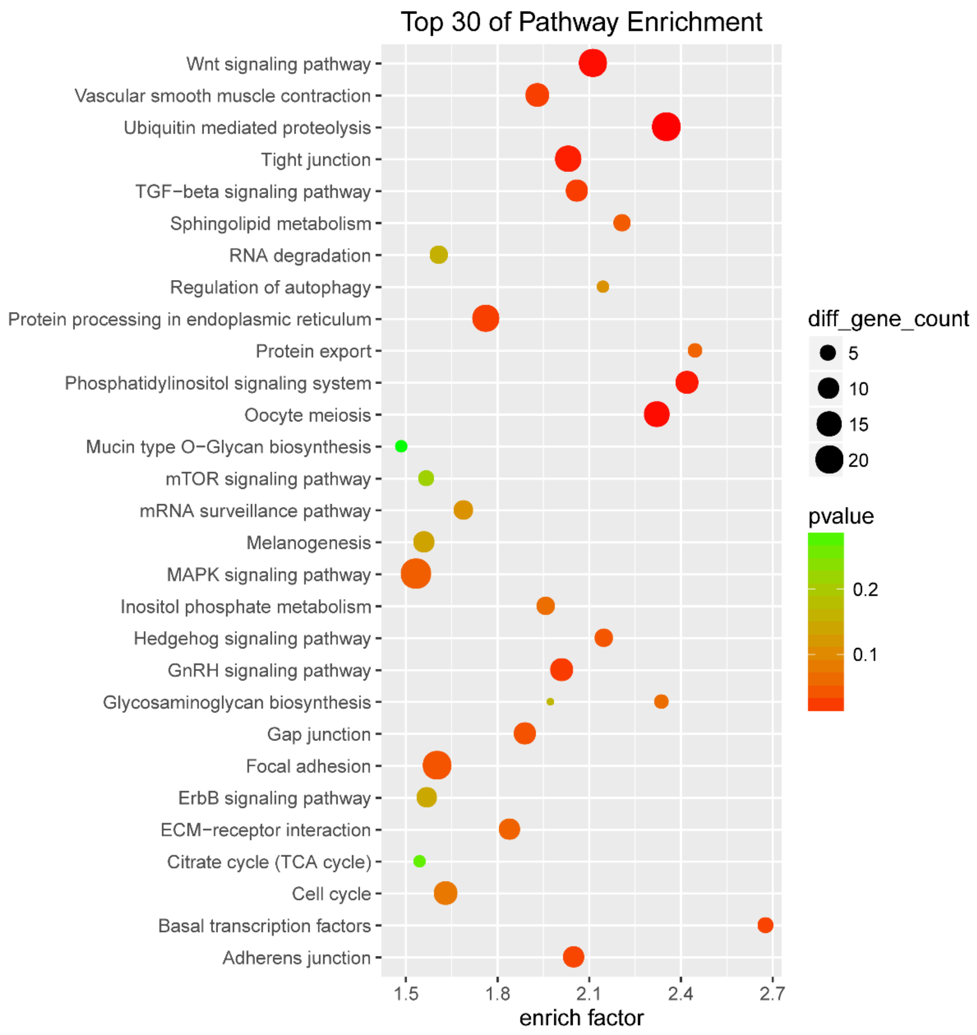

Figure S2. KEGG pathways enrichment analysis of all miRNA target genes.

Table S1 Differentially expressed miRNAs in the three comparisons

miRNA ID	PGCs (TPM)	SSC (TPM)	Spa (TPM)	Sperm (TPM)
miR-184-3p	40.46819763	107.5528328	193.8306668	776.9934324
miR-9-5p	126.7641047	37.13574724	18.06658672	1009.524314
miR-10b-5p	92650.88987	197.253355	279.751992	702207.3419
miR-204	202.3253376	95.151035	122.4046263	816.0636618
miR-211	95.8519725	97.8349489	60.92221102	405.8262534
miR-146a-5p	9.2572853	46.54939974	13.2876566	201.6527967
let-7i	100.253396	9752.337921	427.378302	2239.606374
let-7g-5p	4050.852679	2774.803788	2471.551078	574.78231
miR-458b-5p	50.4547002	54.8102727	6.62246632	221.1879114
miR-10a-5p	94561.20628	79437.42234	92562.33638	18175.2716
let-7a-5p	4070.121988	11480.94988	13094.49399	514.501987
let-7j-5p	3354.713694	3822.461775	4353.627245	446.858817
miR-15b-5p	382.0011438	428.5064966	364.06273	74.4832836
let-7f-5p	9360.65918	12232.93023	22892.25606	882.371765
miR-1454	179.038763	201.6152345	139.3508045	15.1032273
miR-24-3p	1840.489729	2463.566793	2464.129212	402.6754285
miR-181a-5p	5676.43792	3044.36306	5645.8928	47361.9403
miR-429-3p	4084.873928	102.0522355	71.1423951	877.8198308
miR-135a-5p	146.1826256	80.27547757	37.39363297	15.3201931
miR-30c-1-3p	218.5748716	286.6346	217.1491682	38.6362978
miR-101-3p	4893.437293	4570.587717	4056.438896	833.78012
miR-130c-3p	1851.05109	1710.319824	1119.638198	163.914736
miR-148a-3p	35036.43668	34103.21692	44556.7545	8032.80135
miR-30a-3p	623.6236871	985.9520138	766.3035469	136.7458028
miR-153-3p	276.2759502	248.9136052	267.122028	43.4813843
miR-146c-5p	15998.76405	16610.87163	24689.39126	843.539233
miR-19b-3p	1478.370836	1620.77894	1001.715205	77.0760723
miR-142-5p	1139.658003	542.7691141	14.56531022	70.57847886
miR-15a	1681.823107	1486.693246	1663.211794	341.5494245
miR-29b-3p	183.8103364	1106.663354	248.6677433	40.96072434
miR-19a-3p	1404.37128	1430.878441	1261.195227	194.7209818
let-7k-5p	1684.697048	6034.299495	3614.997956	316.3428249
let-7c-5p	6093.651099	3585.637998	4059.338896	503.5018268
miR-219b	11292.41908	2446.734293	2447.182194	555.805521
miR-30c-2-3p	163.3043783	142.0201431	203.0075545	27.09709456
miR-99a-5p	1471.32634	1067.81951	1243.723437	195.3511468
miR-30a-5p	12176.88843	26696.8961	20795.34157	544.470743
miR-2954	14548.82737	7698.144043	16610.48005	1839.451605
miR-10a-3p	1172.12577	6.00741618	1148.174021	68.68798389
miR-1451-5p	192.4321597	188.7996511	155.8803729	36.54956941
miR-29a-3p	1670.405017	1987.099293	1675.116134	264.6692957
miR-202-5p	1133.211054	3070.777918	1408.423483	49.78303419
miR-30b-5p	1694.717005	1970.866302	1149.118946	255.8469859
miR-194	149.8667618	1.60227985	0.777283382	18.2747847

miR-100-5p	8474.087188	19210.67043	16651.09065	310.928321
miR-30e-3p	1840.433586	1960.414924	1958.999632	294.2870502
miR-29c-3p	1399.633161	563.1143149	331.1507309	134.8553078
miR-125b-3p	1836.627556	2369.868485	2707.817216	268.2539296
miR-146b-5p	381.9127041	1709.133852	6106.996489	51.04336417
miR-2131-3p	51.18722118	53.2946153	38.02386274	11.34296982
miR-15c-5p	2294.259411	2713.059123	1819.963522	491.528692
miR-30d	18938.88347	25832.98965	30816.41505	3973.190261
miR-181b-5p	12242.9114	5612.093475	12250.82641	2459.533955
miR-20a-5p	852.473069	715.7340826	676.8667721	166.3635573
miR-128-3p	3312.566649	3734.69985	2703.545659	546.3530462
miR-146c-3p	121.1715706	169.0010054	116.4524562	19.53511468
miR-215-5p	998.3449983	379.2145367	179.1253055	160.6920724
miR-133a-3p	474.7439998	530.8707021	582.8224854	75.61979878
miR-17-3p	60.89648165	69.23111362	48.80779435	9.23524888
miR-23b-3p	192.2433573	108.2199421	77.79836373	28.35742454
miR-181a-3p	1474.254109	800.8275712	1966.94711	203.5432917
miR-18a-5p	372.5249056	192.0533355	124.085239	49.6393419
miR-22-5p	74.48944631	98.28742683	67.57463636	9.671484908
miR-126-5p	603.5276307	66.48855344	45.58661997	78.25347886
miR-1664-3p	53.90581411	28.16683423	34.17245859	6.931814888
miR-34a-5p	165.6788206	245.6444438	379.25827	20.79544466
miR-26a-5p	31743.22315	43140.84218	64284.69691	3855.979573
miR-16-5p	3057.018914	2378.763274	2536.674821	345.9605794
miR-27b-3p	6837.649592	4408.62842	3418.156191	773.2124425
miR-103-3p	5631.293397	4078.705844	3730.680131	618.191855
miR-16-2-3p	51.80861385	53.81347804	39.42437334	5.671484908
miR-16c-5p	326.9302185	302.6452215	203.634241	31.50824949
miR-20b-5p	649.9767327	516.2684222	327.2293013	59.86567403
miR-1552-3p	112.4720733	164.405364	99.15615035	10.25979878
miR-140-3p	4160.612295	3938.687028	5550.85373	368.646519
miR-140-5p	160.2416348	141.4271572	169.3917568	13.86362978
miR-1729-5p	40.39052354	26.46199953	24.50893547	3.31610462
miR-1456-5p	203.4284253	369.133775	383.1096741	15.32296982
miR-107-3p	886.8826881	758.6514431	621.8267056	63.01649898
miR-17-5p	1119.050524	976.7220387	926.0176075	77.25379878
miR-30c-5p	7369.639391	9895.157113	10822.02549	504.7621568
miR-193a-5p	81.24709159	69.67585311	58.68139406	5.5325259
miR-221-3p	3397.542097	6017.547641	3505.267951	216.7767565
miR-100-3p	228.4394803	478.1690728	177.1645907	14.56531022
miR-1684b-3p	79.38291358	93.7659087	115.9622775	5.041319918
miR-365-3p	322.5804698	203.9871785	228.4232786	18.90494969
miR-106-5p	607.8773793	507.225386	315.7451144	35.5824949
miR-92-3p	48800.76264	51446.87098	40034.57581	2806.754865
miR-301b-3p	1882.742116	1230.29767	817.9682149	102.7168933
miR-130a-3p	5094.254782	3483.273793	2129.126237	276.6424305

miR-125b-5p	11051.46863	14359.37797	14857.59678	573.4501407
miR-456-3p	5537.851474	4191.743797	2706.066578	262.1486358
miR-22-3p	121558.3099	178421.9961	144837.935	5682.197713
miR-21-5p	251593.6586	272865.0207	215748.3075	11061.91623
miR-18b-5p	187.8936086	112.8155834	59.73177701	8.192144867
miR-218-5p	1090.932506	469.6448992	546.8993886	46.00204426
miR-92-5p	45.43933899	52.99812231	42.57552218	1.785616499
miR-2131-5p	170.4946138	151.8785351	99.08612482	5.785659959
miR-126-3p	238.9254816	20.68038619	16.385974	7.780989939
miR-460a-5p	221.2157905	233.3399847	89.00244851	6.780989939
miR-18a-3p	84.35405494	48.03186469	31.58151399	2.520659959
miR-27b-5p	64.70251175	44.1774558	34.94273942	1.890494969
miR-455-5p	1461.127189	939.2156752	916.564161	39.07022937
miR-1552-5p	118.3753036	166.4066917	112.8111287	3.150824949
miR-455-3p	268.5193075	199.3174138	233.0449635	7.041319918
miR-147	150.299352	1548.360527	474.3529396	3.780989939
miR-148a-5p	306.2689122	573.8621859	342.8449944	7.35268064
miR-3523	407.7889396	566.3016146	342.0747136	9.452474847
miR-106-3p	383.1662551	394.9286653	247.1901206	8.32334888
miR-31-5p	4814.783429	812.3907979	367.1438533	99.56606839
miR-193b-3p	389.0694854	261.6550654	131.8580728	7.561979878
miR-130b-3p	3817.603542	2903.704117	3385.734371	57.97517906
miR-128-1-5p	88.62612955	132.9029836	6.76302798	1.26032998
miR-383-5p	112.0060288	109.9247768	64.49351304	1.52016499
miR-130b-5p	627.2959003	568.7476818	699.4850182	7.5825898
miR-196-5p	3343.247912	389.2211752	694.4431801	33.39874446
miR-214	361.2621635	376.6943463	740.4499532	3.32132998
miR-21-3p	2199.030985	2253.569109	1304.715673	15.75412474
miR-222a	11524.89217	25498.76792	11451.69506	54.19418912
miR-221-5p	328.328352	593.5789699	216.6589895	0.93016499
miR-1559-5p	3677.401821	3970.634148	2045.795856	8.25316499
miR-301a-5p	265728.425	35212.7152	18523.11338	578.92542
miR-193a-3p	387.3606556	361.9438199	376.6673254	0.7816499
miR-301a-3p	2805.587905	1473.421923	1079.443544	4.691710504
miR-199-3p	7636.061498	8134.804097	9224.883208	10.08263984
miR-130a-5p	202.4963363	220.5166628	191.7999264	0.18016499
miR-199-5p	1658.18634	1449.924854	2345.645175	0.653134
miR-454-3p	14347.72373	11455.00674	14903.11338	4.271557324
